# Intravenous administration of human amnion-derived mesenchymal stem cells improves gait and sensory function in mouse models of spinal cord injury

**DOI:** 10.3389/fcell.2024.1464727

**Published:** 2024-09-11

**Authors:** Shoichiro Tsuji, Yoji Kuramoto, Saujanya Rajbhandari, Yuki Takeda, Kenichi Yamahara, Shinichi Yoshimura

**Affiliations:** ^1^ Department of Neurosurgery, Hyogo Medical University, Hyogo, Japan; ^2^ Laboratory of Molecular and Cellular Therapy, Institute for Advanced Medical Sciences, Hyogo Medical University, Hyogo, Japan

**Keywords:** human amnion-derived stem cell, mesenchymal stem cell, spinal cord injury, inflammation, neurotrophic factors, serum nitric oxide

## Abstract

**Introduction:**

Spinal cord injury (SCI) leads to severe disabilities and remains a significant social and economic challenge. Despite advances in medical research, there are still no effective treatments for SCI. Human amnion-derived mesenchymal stem cells (hAMSCs) have shown potential due to their anti-inflammatory and neuroprotective effects. This study evaluates the therapeutic potential of intravenously administered hAMSCs in SCI models.

**Methods:**

Three days after induction of SCI with forceps calibrated with a 0.2 mm gap, hAMSCs or vehicle were administered intravenously. Up to 4 weeks of SCI induction, motor function was assessed by scores on the Basso Mouse Locomotor Scale (BMS) and the Basso-Beattie-Bresnahan Scale (BBB), and sensory function by hindlimb withdrawal reflex using von Frey filaments. Six weeks after SCI induction, gait function was assessed using three-dimensional motion analysis. Immunohistochemistry, polymerase chain reaction (PCR), flow cytometry, and ELISA assay were performed to clarify the mechanisms of functional improvement.

**Results:**

The hAMSC treatment significantly improved sensory response and gait function. In the SCI site, immunohistochemistry showed a reduction in Iba1-positive cells and PCR revealed decreased TNFα and increased BDNF levels in the hAMSC-treated group. In assessing the systemic inflammatory response, hAMSC treatment reduced monocytic bone marrow-derived suppressor cells (M-MDSCs) and Ly6C-positive inflammatory macrophages in the bone marrow by flow cytometry and serum NO levels by ELISA assay.

**Discussion:**

This study demonstrates the therapeutic potential of the hAMSC in SCI, with improvements in gait and sensory functions and reduced inflammation both locally and systemically. The findings support further investigation of the hAMSC as a potential treatment for SCI, focusing on their ability to modulate inflammation and promote neuroprotection.

## Introduction

Spinal cord injury (SCI) is one of the most common and severe central nervous system injuries, which often results in significant neurological dysfunction and disability (Bottai et al., 2010). This imposes a substantial socioeconomic burden on the patient, their family, and society. The pathophysiology of SCI involves primary injury, which causes immediate structural damage, followed by a series of secondary injuries, comprising hemorrhage, edema, demyelination, and axonal and neuronal necrosis (Tator and Fehlings, 1991). To date, no effective treatment has been satisfactory in intervening in this complex pathway involved in tissue damage, leaving the patient handicapped (Ahuja et al., 2017).

In recent years, stem cell transplantation has shown a remarkable therapeutic efficacy on SCI. The therapeutic effects of stem cells are attributed to their pleiotropic, anti-inflammatory, and neurotrophic effects (Lovell-Badge, 2001; Dang et al., 2015). Bone marrow mesenchymal stem cells have shown beneficial effects on the outcomes of SCI (Bottai et al., 2010; Oh et al., 2015; Muniswami et al., 2019). However, their application is limited because of ethical issues, potential hazards for bone marrow donors (e.g., bone marrow aspiration), inadequate sources, immunogenicity, and the low survival rate of transplanted cells (Baxter et al., 2004; Hess, 2009). Moreover, the isolation of these cells also requires highly invasive procedures compared with iPS cells, and their differentiation potential decreases with increasing age (Stolzing and Scutt, 2006; Deng et al., 2018).

Human amnion-derived mesenchymal stem cells (hAMSCs) are an attractive alternative source, as they are readily obtained from the placenta and are abundant in the amnion. hAMSCs do not have ethical issues or legal concerns and have an advantage over other stem cells in terms of renewal, multi-differentiation potential, no tumorigenicity, less immunogenicity, and immunomodulatory effects (Abbasi-Kangevari et al., 2019; Magatti et al., 2018). Furthermore, several studies have shown the efficacy of hAMSCs in treating neurological diseases such as cerebral ischemia (Li et al., 2012), intracerebral hemorrhage (Kuramoto et al., 2022a), traumatic spinal cord injury (Zhou et al., 2016), and Alzheimer’s disease (Zheng et al., 2017). These characteristics make them a promising source of stem cells for cell therapy in SCI. However, the therapeutic efficacy of hAMSCs in the recovery of sensory, motor, and gait functions in an SCI mice model is unknown and less explored.

Our study aims to examine the therapeutic effect of intravenously injected hAMSCs on SCI mice. To address this hypothesis, we induced SCI in mice, treated them with hAMSCs, and evaluated their neurofunctional recovery (sensory, motor, and gait). We performed histological immunostaining, flow cytometry, measurement of serum NO concentration, and polymerase chain reaction (PCR) to understand the potential underlying mechanism of hAMSC treatment.

## Materials and methods

### Cell preparation of hAMSCs

Our Institutional Ethics Committee approved this study (approval numbers: 325). The cell preparation procedures of hAMSCs have been described previously (Kuramoto et al., 2022a; Yamahara et al., 2014). Briefly, we first obtained written informed consent for obtaining hAMSCs from the donors: pregnant women waiting for Cesarean section. Regarding hAMSCs, human fetal membranes were obtained by Cesarean section. Amnia were detached mechanically from the chorion and digested with collagenase/dispase solution for 1 h at 37°C in a water bath shaker. The cells were filtered through a 100-μm mesh filter, re-suspended in α-minimal essential medium (α-MEM; Invitrogen, CA) supplemented with 10% bovine-derived platelet lysate “NeoSERA” (Japan Biomedical Co., Ltd, Japan), plated on dishes, and incubated at 37°C with 5% CO_2_. Gentamicin was used in the primary culture but not in subsequent cultures. Spindle-shaped cells formed visible colonies in 1–2 days.

### Mice and SCI induction

All experiments were approved and confirmed by our institutional animal care ethical committee (approval numbers: 19-048 and 23-060A). The procedure has been described previously (Kuramoto et al., 2022b). Briefly, 7- to 9-week-old male C57BL/6J mice were housed under controlled 12-h light cycling conditions and had free access to water and food (CLEA Japan, Inc, Tokyo, Japan). SCI was induced in mice models under general anesthesia with inhalant isoflurane (1.5%–2%) by using a surgical microscope at the Th9–Th10 vertebral levels by using calibrated forceps with a gap of 0.2 mm (Dumont: Inox No.3 forceps). The reliability of the calibrated forceps compression model of SCI for generating reproducible injuries has been demonstrated in a previous study (McDonough et al., 2015). At first, the mice were placed in a prone position, and laminectomy was carried out to expose the spinal cord adequately. Careful compression of the spinal cord was carried out with calibrated forceps with persistent pressure for 20 s. Hemostasis was ensured, and the wound was closed using sutures.

### hAMSC administration

We randomly assigned SCI mice into two groups (hAMSC group and untreated SCI group. In the hAMSC group, intravenous administration of 10^5^ hAMSCs with 50 uL vehicle from the subclavian vein was carried out on day 3 post-SCI. The untreated SCI group received intravenous administration of the same volume (50 µL) of physiological saline intravenously.

### Neurobehavioral tests

Recovery of the SCI mice was evaluated with hind limb motor and sensory examination and gait analysis. The hind limb’s motor function was assessed using the Basso mouse locomotor scale (BMS) and the Basso–Beattie–Bresnahan (BBB) score. The BMS score is a 0 (complete paralysis) to 9-point scale (normal gait), while the BBB score is a 0 (complete paralysis) to 21-point scale (normal gait) (Basso et al., 2006; Basso et al., 1996). Both scoring systems are developed to describe the recovery of locomotor function in thoracic SCI mice. The effect of hAMSC administration on the recovery of BMS score and BBB score was examined on the day of transplantation and once weekly thereafter for 4 weeks. Scores were recorded for each SCI mouse by three observers for the left and right hind limbs and averaged to get a single calculated value per mouse per test. Sensory examination of the bilateral hind paw region of SCI mice was done by examining the paw withdrawal reflex after the application of a series of forces using TACTILE TEST (AESTHESIO) Semmes–Weinstein von Frey anesthesiometer (Muromachi Co. Ltd, Japan) (Godfrey et al., 2018; Ranade et al., 2014). The examination was done before induction of SCI, on the day of transplantation (day 3), and once weekly thereafter for 4 weeks. A series of 20 von Frey filaments with increasing calibrated forces from 0.008 g to 300 g were used. Each filament was directed at the center of the plantar paw and pressed upward until the filament bent. Withdrawal of the paw after the application of the force was considered a positive response. The calibrated force of the filament that generated the positive response was recorded for each SCI mouse model for the left and right hind paws and averaged to get a single calculated force per mouse per test.

### Gait analysis

Gait performance was evaluated in the fifth week post-SCI using a KinemaTracer^®^ three-dimensional treadmill gait analysis system (Kissei Comtec Co., Ltd., Matsumoto, Japan) and a computer for recording and data analysis. Previous studies have verified the reliability of this system for gait analysis (Pongpipatpaiboon et al., 2016). The system consisted of four charge-coupled cameras installed around a treadmill and a computer-controlling system. Three colored markers were placed bilaterally on knee joints, lateral malleoli, and the fifth metatarsal heads, and they were subjected to walking on the treadmill. The video system recorded walking. We obtained a more in-depth look at the motion, such as measuring gait speed, rate, and toe height for each SCI mouse. We compared the obtained data between the hAMSC and untreated SCI groups using computer software (KinemaTracer software).

### Immunostaining

The procedure has been described previously. Mice were divided into two groups: the hAMSC group that received an intravenous injection of 1.0 × 10^5^ hAMSCs in 50 μL of vehicle control at 72 h after the SCI induction and the ICH group that received an intravenous injection of 50 μL of vehicle control alone. The mice were sacrificed on Day 6 after the SCI inductions. The mice were perfused with normal saline and 4% paraformaldehyde in PBS under deep anesthesia with 3%–4% isoflurane. After removal, the spine tissues were fixed with 4% paraformaldehyde for 1 day and then transferred to 30% sucrose solution at 4°C. The spine tissue was sectioned in 8-μm thickness by using a cryostat (CM1950, Leica Biosystems). The primary antibodies used in this experiment were anti-Iba1 (ab178847, Abcam) at 1:250 dilution. The second antibodies used were Alexa 488-labeled anti-rabbit (CST) and Alexa 555-labeled anti-mouse (CST). The immunostaining assay was visualized by using a fluorescence microscope (BZ-x710, Keyence).

### RNA isolation and reverse transcription-polymerase chain reaction (RT-PCR)

The procedure has been described previously (Kuramoto et al., 2022b). Briefly, SCI tissues were extracted from the hAMSC and untreated SCI models post-SCI for 7 days and post-treatment for 3 days and soaked in RNA later stabilization solution (Thermo Fisher Scientific). The total RNA was extracted using TRIzol reagent (Thermo Fisher Scientific). Then, the cDNA was synthesized from the total RNA using ReverTra Ace qPCR RT Master Mix with gDNA Remover (TOYOBO) and THUNDERBIRD SYBR qPCR Mix (TOYOBO). GAPDH mRNA was amplified from the SCI samples, and GAPDH was used as an internal control. Primers used in this study are listed in Supplementary Table S1. After initial denaturation at 95°C for 1 min, a two-step cycle procedure was conducted (denaturation at 95°C for 15 s, annealing and extension at 60°C for 15 s, and data collection at 72°C for 35 s) for 40 cycles using a 7500 Sequence Detector and 7500 software ver.2.0.6 (Applied Biosystems, CA). Afterward, the samples were subjected to electrophoresis, fluoresced, and photographed, and the signal intensity was measured using ImageJ. RNA expression levels of the untreated SCI group on Day 7 were used as the control.

Quantitative real-time RT-PCR (qPCR) was performed with the same sample. The levels of the target gene (BDNF, TSG-6, and TNF) were calculated by the ΔΔCT method. Results were normalized with GAPDH expression.

### ELISA of nitric oxide (NO)

NO is an essential biological messenger. Increased serum NO concentration is observed in the blood in many inflammatory diseases. Therefore, we measured the serum NO concentration between normal (untreated and no SCI, n = 4), hAMSC-treated (n = 5), and untreated SCI group (n = 5) using the Griess Reagent System (Catalog NoG2930, Promega).

### Flow cytometry

The procedure has been described previously (Kuramoto et al., 2022a). Briefly, we prepared hAMSC-treated SCI (n = 12), untreated SCI (n = 13), and normal (n = 6, untreated and no SCI) mice. These mice were transcardially perfused with PBS under deep anesthesia with 3%–4% isoflurane, and the bone marrow was extracted. The bone marrow was washed, re-suspended in 25% Percoll (GE Healthcare), and centrifuged for 20 min at 500 g. The supernatants were discarded. The cell pellets were re-suspended in Histopaque (Sigma) and centrifuged for 20 min at 500 g. The cells were collected and incubated with the following antibodies: APC Rat anti-mouse Ly-6C clone: AL-21 (BD Pharmingen), FITC Rat anti-mouse Ly-6G clone:1A8 monoclonal antibody (BD Pharmingen), PE anti-mouse CD49b (pan-NK cells) Antibody clone: DX5 (BioLegend), PE anti-mouse CD90.2 (Thy1.2) Antibody clone:30-H12 (BioLegend), PE anti-mouse/human CD45R/B220 Antibody clone: RA3-6B2 (BioLegend), PE anti-mouse NK-1.1 Antibody clone: S17016D (BioLegend), PerCP-Cy™5.5 Rat Anti-CD11b clone: M1/70 (BD Pharmingen), Purified Rat Anti-Mouse CD16/CD32 (Mouse BD Fc Block™: BD Pharmingen), and Zombie Yellow™ Fixable Viability Kit (BioLegend). The fluorescent-labeled cells were analyzed by LSRFortessa X-20 (BD Biosciences) and BD FACSDiva software (BD Biosciences). Flow cytometry results were analyzed on FlowJo software version 10.5 (BD Bioscience).

### Statistical analysis

All the results were expressed as mean ± SEM. Statistical data analysis used the Wilcoxon test for two-group comparisons and the Steel–Dwass test for three-group comparisons. The significance level was set at *P <* .05 (two-tailed). These statistical analyses were conducted on JMP version 16 (SAS Institute).

## Results

### The BMS and BBB scales revealed that both groups were equally impaired before hAMSC administration and showed spontaneous recovery over time

**FIGURE 1:**
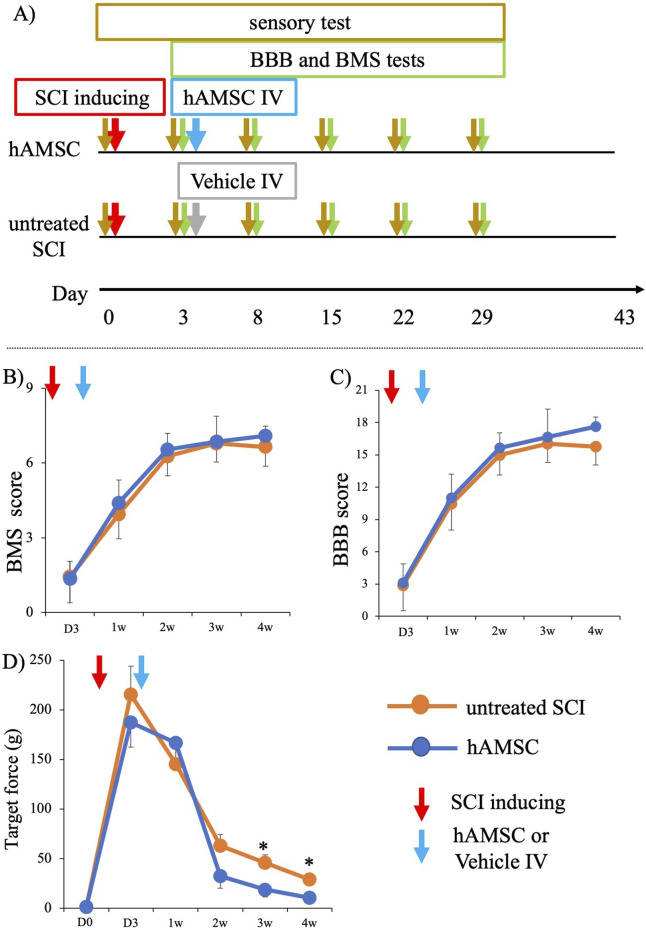
Functional analysis of SCI mice after administration of hAMSC **(A)**. Although there was no difference in motor function among the two groups, significant improvement in sensory function was observed in the hAMSC-treated SCI mice. After hAMSC administration 3 days post-SCI, no differences in BMS score and BBB score were observed between the hAMSC-treated (n = 7) and vehicle (n = 6) group up to week 4 after SCI **(B, C)**. A significant increase in paw withdrawal reflex on the application of lighter force with a von Frey filament was observed in the third week and fourth week after SCI in the hAMSC-treated (n = 7) group compared to the untreated SCI group (n=6) **(D)**. P values are based on the Wilcoxon test and compared with those of the untreated SCI group. *P < 0.05 compared with the untreated SCI group.

To evaluate the therapeutic efficacy of hAMSCs for SCI, we induced SCI and treated the SCI mice with intravenous administration of hAMSCs (hAMSC group; n = 7) or vehicle (untreated SCI group: n = 6) after 3 days of SCI induction. First, BMS and BBB tests were conducted according to our protocol (Figure 1A). BMS scores of both groups were similarly impaired before treatment: untreated SCI group = 1.47 ± 1.13 and hAMSC group = 1.36 ± 0.71. BMS scores were improved gradually in both groups. After 1 week: untreated SCI group = 3.95 ± 1.08, hAMSC group = 4.41 ± 0.91; 2 weeks: untreated SCI group = 6.27 ± 0.86, hAMSC group = 6.55 ± 0.64; 3 weeks: untreated SCI group = 6.78 ± 0.82, hAMSC group = 6.86 ± 1.02; 4 weeks: untreated SCI group = 6.65 ± 0.85, hAMSC group = 7.10 ± 0.39, and it gradually improved over time in both groups. BBB scores were also similarly impaired before treatment: untreated SCI group = 2.94 ± 2.59 and hAMSC group = 3.09 ± 1.80. After 1 week: untreated SCI group = 10.5 ± 2.66, hAMSC group = 11.00 ± 2.21; 2 weeks: untreated SCI group = 15.00 ± 2.04, hAMSC group = 15.67 ± 1.37; 3 weeks: untreated SCI group = 16.47 ± 1.88, hAMSC group = 16.67 ± 2.60; 4 weeks: untreated SCI group = 16.22 ± 1.90, hAMSC group = 17.62 ± 0.91. There were no significant differences in BBB and BMS scores at any period.

### hAMSC administration led to a decrease in the escape behavior threshold

In contrast, the SCI mice that were treated with hAMSCs showed marked improvement in sensory function after 3 weeks: untreated SCI group = 45.92 ± 8.03 g, hAMSC group = 18.71 ± 7.40 g, P *< 0.05*), and at 4 weeks: untreated SCI group = 28.91 ± 5.16 g, hAMSC group = 10.57 ± 2.93 g, P *< 0.05*. This was indicated by a significant increase in paw withdrawal reflex when lighter force was applied to a von Frey filament (Figure 1D).

### hAMSC administration caused improvement of gait function in a three-dimensional gait analysis

**FIGURE 2:**
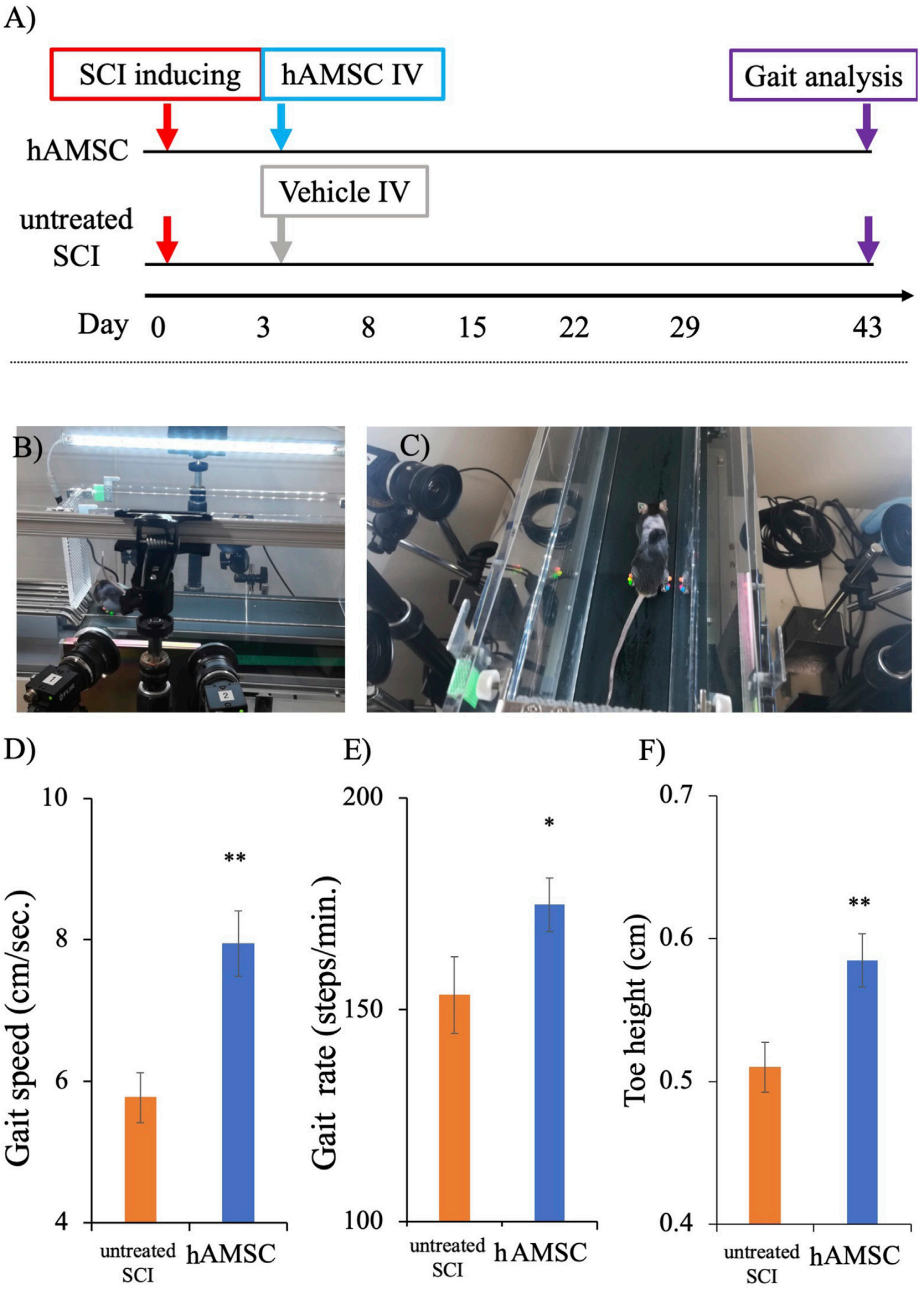
Gait analysis was done on the 43rd day post-SCI using a KinemaTracer^®^ three-dimensional treadmill gait analysis system (Kissei Comtec Co., Ltd., Matsumoto, Japan) **(A)**. Picture demonstrating the KinemaTracer^®^ three-dimensional treadmill gait analysis system consisting of four charge-coupled cameras installed around a treadmill **(B)**. A SCI mouse model with markers placed in the hind limb is subjected to walking on the treadmill, while the camera simultaneously captures the gait of the mice **(C)**. Comparison of hind limb gait among hAMSC-treated and vehicle groups **(D–F)**. There was a significant improvement in gait speed, gait rate, and toe height in the hAMSC-treated SCI mice **(D–F)** (*P < 0.05; **P < 0.01). P values are based on the Wilcoxon test and compared with the untreated SCI group.

We conducted a three-dimensional gait analysis to investigate motor function, which is more detailed and challenging to detect in BBB and BMS scores. The gait analysis data for the two groups at 6 weeks (43 days) after SCI were obtained from the KinemaTracer^®^ three-dimensional treadmill gait analysis system (Kissei Comtec Co., Ltd., Matsumoto, Japan) (Figures 2A–C). The hAMSC-treated SCI mice exhibited an increase in gait speed: untreated SCI group = 5.77 ± 0.49 cm/sec, hAMSC group = 7.94 ± 0.40 cm/sec (*P < 0.01*; Figure 2D); gait rate: untreated SCI group = 153 ± 8.98 steps/min, hAMSC group = 175 ± 6.34 steps/min (*P < 0.05*; Figure 2E); and toe height: untreated SCI group = 0.50 ± 0.017 cm, hAMSC group = 0.58 ± 0.019 cm (*P < 0.01*; Figure 2F).

### hAMSC administration suppressed the expression of Iba1-positive cells around SCI

**FIGURE 3:**
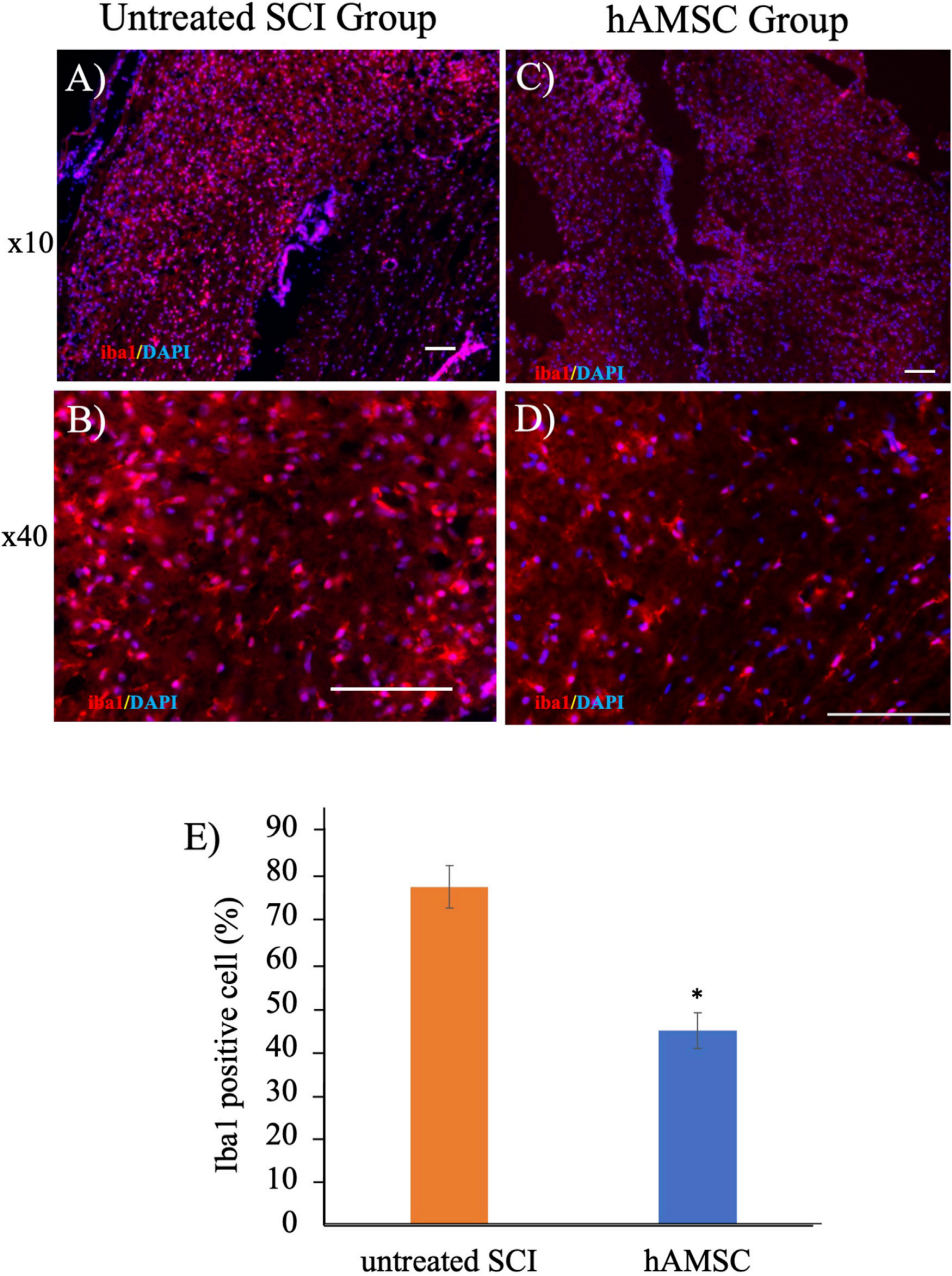
Iba1-positive cells decreased in the hAMSC group **(C, D)** compared with the untreated SCI group **(A, B)** at the SCI site. (**(E)**; *P < 0.05); n = 5 in the hAMSC group and n = 4 in the untreated SCI group. P values are based on the Wilcoxon test and compared with the untreated SCI group.

To elucidate the mechanism of a series of reactions occurring within the actual tissue, we extracted the spinal cord injury site and performed histological immunostaining a week after the injury. Focusing on the IBA1, expressed explicitly in macrophages, staining revealed significant suppression of Iba1 in the hAMSC group compared to the untreated SCI group (Figure 3).

### hAMSC administration suppressed the mRNA expression of TNFα and promoted the mRNA of BDNF expression around SCI

**FIGURE 4:**
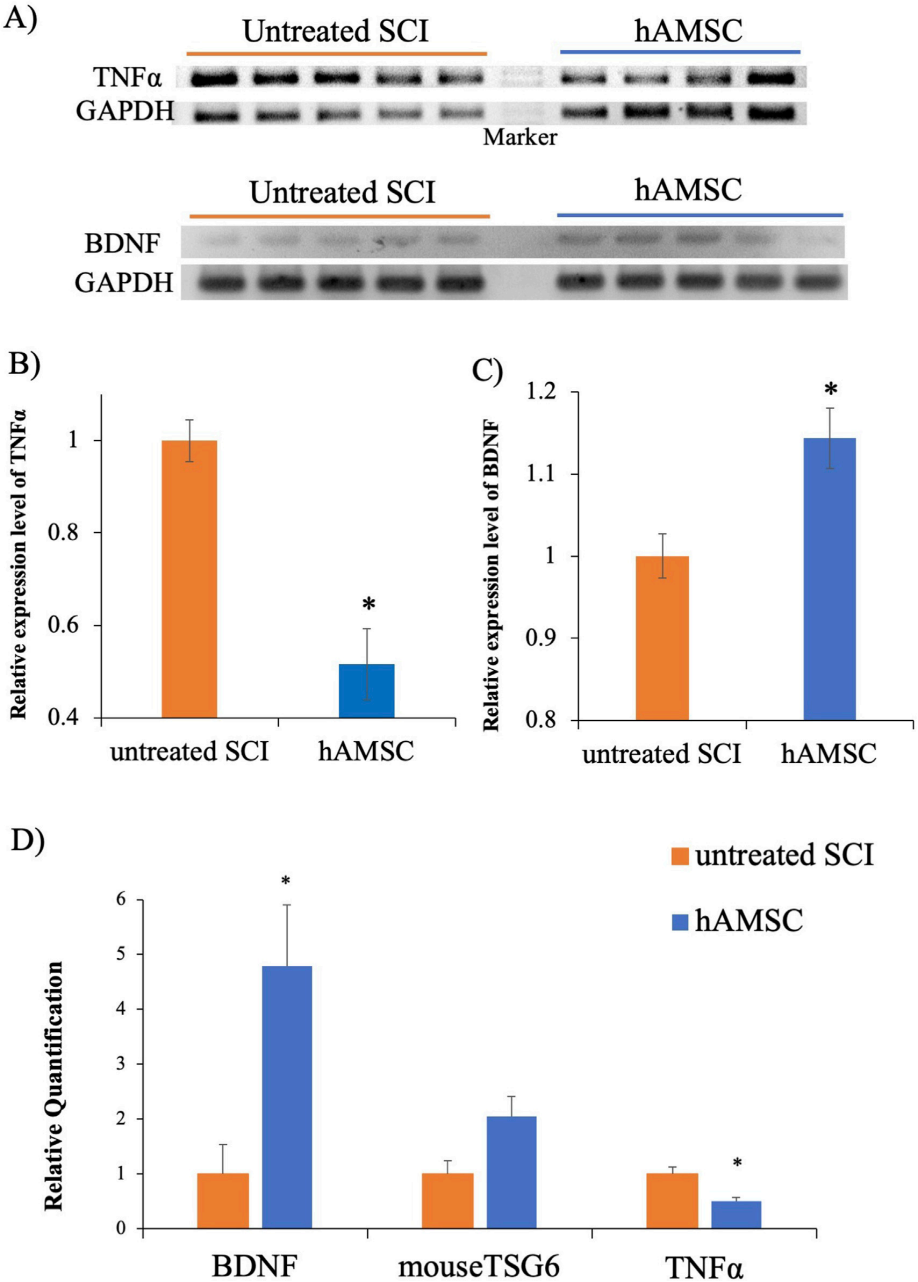
PCR at SCI site **(A)**, the hAMSC treatment suppressed TNFα expression **(B)** and enhanced BDNF expression **(C)** n = 5 in untreated SCI and n = 4–5 in hAMSC groups. Quantitative PCR at the SCI site, where the hAMSC treatment suppressed TNF-α expression and enhanced BDNF expression **(D)**. P values are based on the Wilcoxon test. *P < 0.05 compared with the untreated SCI group.

To confirm the functional changes at the SCI, we examined whether mRNA expression associated with inflammation and neuroprotection was changed by hAMSC treatment in untreated SCI (n = 5) and in hAMSC (n = 4–5) groups. The hAMSC treatment suppressed the relative expression of TNFα (0.52 ± 0.08; *p = 0.02*, Figure 4B) and enhanced the relative expression of BDNF (1.14 ± 0.04; *P = 0.047*, Figure 4C) at the SCI. In qPCR, the hAMSC treatment suppressed the relative quantification of TNFα (0.51 ± 0.06; *P = 0.015*, Figure 4D) and enhanced the relative quantification of BDNF (4.78 ± 1.12; *P = 0.025*, Figure 4D) at the SCI. The quantification of TSG6 showed an increasing trend with hAMSC treatment, but no significant difference was observed (2.04 ± 0.37, *P = 0.10*, Figure 4D). The results confirm that hAMSC treatment produced an anti-inflammatory and neuroprotective effect at the SCI site.

### hAMSC administration suppressed the serum nitric oxide (NO) concentration

**FIGURE 5:**
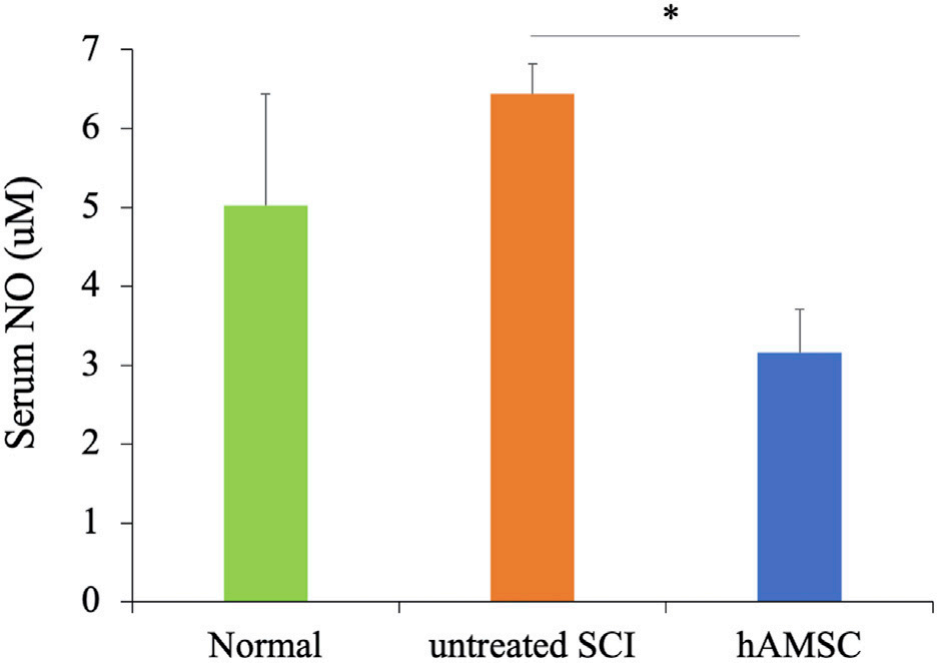
The serum NO concentration was measured 6 days after the injury using the ELISA kit. It was significantly suppressed compared to the untreated group n = 5 each in untreated SCI and hAMSC groups and n = 4 in the normal group. P values are based on the Steel–Dwass test. *P < 0.05.

Anti-inflammation and neuroprotective effects were observed in SCI, but whether these responses occurred only at the focal site of the SCI or the whole body was unclear. So we focused on the serum NO concentration to confirm that. NO, released from activated microglia during inflammation, induces neurotoxicity and neuronal cell death. Therefore, increased serum NO concentration suggests an exacerbation of inflammatory reactions. We collected the serum 7 days after tissue injury and measured the serum NO concentration. The serum NO concentration was significantly decreased in the hAMSC group (16 ± 0.5 uM, *P = 0.024*) compared to the untreated SCI group (26 ± 5.0 uM, Figure 5). This result suggests that the activation and exacerbation of inflammatory reactions due to tissue injury are suppressed in the hAMSC group.

### hAMSC administration suppressed monocytic-myeloid-derived suppressor cells (M-MDSCs) and non-lymphocytic inflammatory M1 macrophages in the bone marrow

We performed another investigation to examine whether there is a systemic inflammatory response. We focused on the flow cytometry of immune cells in the bone marrow. The bone marrow produces the blood cells and has an essential role in the immune response, and we investigated the MDSCs, which account for 20%–30% of the immune cells in the bone marrow (Gabrilovich and Nagaraj, 2009).

**FIGURE 6:**
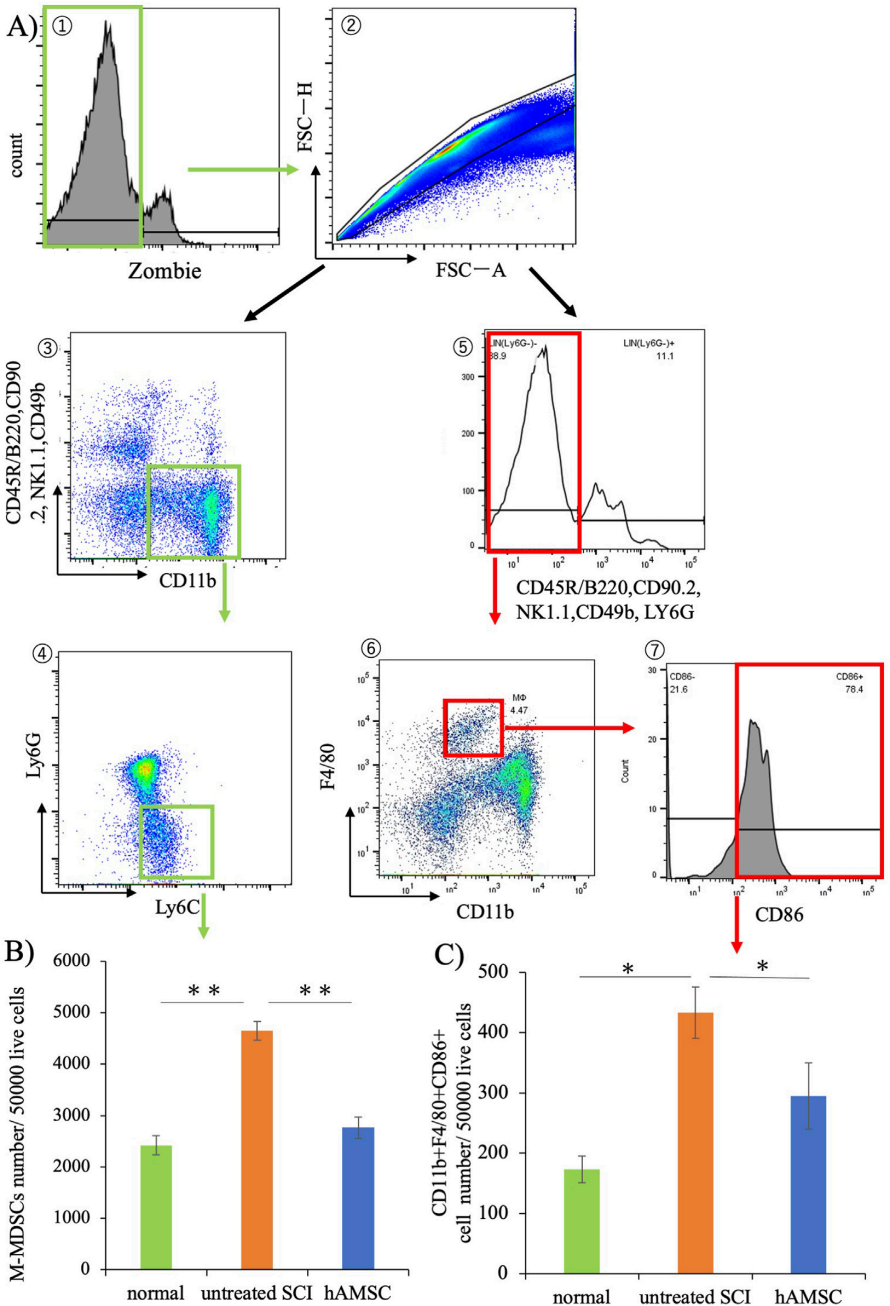
Results of bone marrow cell flow cytometry. **(A)** Gating strategy. **(B)** Significant suppression of M-MDSCs was observed in the hAMSC group compared to the untreated SCI group. **(C)** Non-lymphocytic Ly6G^−^CD11b^+^F4/80^+^Ly6C^+^ cells were also significantly suppressed compared to that in the untreated SCI group. n = 6 in normal group, n = 13 in the untreated SCI group, and n = 12 in the hAMSC group. P values are based on the Steel–Dwass test. *P < 0.05,**P < 0.01.

Seven days post-SCI, bone marrow was harvested from normal, untreated SCI, and hAMSC groups and subjected to flow cytometry (Figure 6A). The results revealed an increase in monocytic-myeloid-derived suppressor cells (M-MDSCs), implicated in immune cell regulation, in the untreated SCI group (4,650± 433 cells, *P = 0.012*) compared with the normal group (2,420± 187 cells, Figure 6B). In contrast, M-MDSCs were significantly more suppressed in the hAMSC-treated group (2,770± 210 cells, *P < 0.001*) than in the untreated SCI group (Figure 6B). Additionally, non-lymphocytic Ly6G^−^CD11b^+^F4/80^+^Ly6C^+^ cells, so-called inflammatory M1 macrophages, were also significantly suppressed in the hAMSC group (290 ± 55 cells, *P = 0.044)* than in the untreated SCI group (430 ± 42 cells, Figure 6C). These findings in the bone marrow paralleled the reduced macrophage activity at the site of spinal cord injury, which could be related to the regulation of the inflammatory response.

## Discussion

This study demonstrated that intravenous hAMSC treatment in the acute phase for SCI mice improved the functional outcomes in the chronic phase. While the administration of hAMSCs did not result in differences in the recovery of BMS and BBB scores (Figures 1B, C), a notable improvement in the sensory function at the chronic phase was observed in the hAMSC-treated group (Figure 1D). Furthermore, the gait analysis revealed that hAMSC administration led to improvement in gait speed, gait rate, and toe height (Figure 2).

The hAMSC treatment suppressed the local accumulation of macrophages at SCI (Figure 3). In addition, PCR analysis of the SCI tissue extracted 3 days after injection revealed localized suppression of TNFα production and enhancement of BDNF expression due to hAMSC treatment (Figure 4). The serum NO concentration, which typically increases during inflammatory responses, was significantly suppressed in the hAMSC treatment group (Figure 5), suggesting a systemic response. Additionally, flow cytometry of bone marrow revealed suppressed production of M-MDSCs (Figure 6B), and production of M1 macrophages was also inhibited by hAMSC treatment (Figure 6C).

Based on these results, it is inferred that hAMSC treatment may mitigate exaggerated local and systematic inflammatory responses caused by spinal cord injury and may have the potential to produce neuroprotective effects. This suggests the potential improvement in gait and sensory function in the chronic phase. We performed the procedure along the rigid schedule (Figure 1A). We evaluated the motor sensory function by BMS and BBB scores, which have been widely used as locomotor estimating scales. BMS was determined from frequency analyses of seven locomotor categories to estimate the locomotor function more precisely (Basso et al., 2006). BBB offers investigators a more discriminating measure of behavioral outcomes to evaluate treatments after SCI (Basso et al., 1995) (Figures 2B, C). In our experiments, the degree of SCI in mice targeted complete bilateral paraplegia and incomplete bilateral paraplegia. They were classified as A or B on the Asian Impairment Scale, a modified version of the Frankel classification in humans (Alexander et al., 2009). The purpose was to expand the range of clinical indications, and therefore, the SCI model was also designed to avoid complete spinal cord amputation. The most severe pretreatment symptoms from this BMS and BBB were nearly equivalent, indicating that the two groups were equally impaired. On the other hand, these scores were subjective to the observer, and the sensitivity to distinguish symptoms decreased with improvement. For this reason, they were not considered appropriate for evaluating the effect of hAMSCs. So we introduced a three-dimensional treadmill gait analysis system to evaluate the gait function. The results and previous publications utilizing kinematic analyses provide strong evidence supporting the use of kinematic analyses in both rodent experiments and clinical studies (DiGiovanna et al., 2016; Kawai et al., 2015; Miri et al., 2017; Ueno and Yamashita, 2011; Ueno et al., 2018), which was very effective in detecting the subtle discrepancy. As far as our investigation extends, a three-dimensional treadmill gait analysis system is the most sophisticated method. In our experiments, a significant improvement was observed in three categories.

As for the sensory function, it was evaluated using the paw withdrawal reflex. Inflammation is recognized as a significant factor in the pathophysiology of SCI (Sochocka et al., 2017). While inflammation is a vital defense mechanism, prolonged or excessive inflammatory responses from microglia can lead to neural damage and are associated with various CNS conditions (Sochocka et al., 2017). Macrophages and microglia are pivotal cellular components participating in the inflammatory process, potentially exacerbating secondary SCI and neurological impairment through the secretion of pro-inflammatory cytokines and other harmful substances (Hausmann, 2003). Suppressing activated macrophages/microglia may improve tissue damage and facilitate functional recovery in SCI (Tian et al., 2007; Popovich et al., 1999). In the present study, we found some results supporting the idea that anti-inflammation may lead to a good outcome induced by hAMSC injection. First, macrophages at the focal sight of SCI decreased, reflected by the number of iba1-positive cells (Figure 3). The inflammatory response following SCI is intricate and regulated by various cell types and numerous inflammatory cytokines, including tumor necrosis factor-alpha (TNFα), interleukin 1β (IL-1β), and interleukin 6 (IL-6). While inflammation after SCI can have some beneficial effects, the excessive infiltration of immune cells is a primary driver of neural degeneration (Glaser et al., 2004; Garcia et al., 2016). These immune cells migrate to the injury site from the peripheral tissues under cytokines and chemokines released by microglia, astrocytes, and peripherally derived macrophages within the lesion area (García et al., 2016; Mortazavi et al., 2015). In the mice intracerebral hemorrhage model, a lesion similar to SCI, hAMSC treatment had suppressed TNFα expression in the damaged site (Kuramoto et al., 2022a). Therefore, the mechanisms that facilitate the damage caused by SCI were investigated in the current study from the perspective of inflammation suppression and cell protection.

Second, we focused on MDSCs, which were initially characterized in cancer patients and linked to enhanced tumor progression and impaired T-cell function (Bronte et al., 2000). However, MDSCs are not limited to cancer and also contribute to various other pathological conditions characterized by inflammation, including chronic infections, autoimmunity, asthma, and obesity (Dorhoi and Du Plessis, 2017; Cripps and Gorham, 2011; Deshane et al., 2015; Clements et al., 2018). MDSCs are also a diverse group of immature myeloid cells originating from hematopoietic precursor cells in the bone marrow (Veglia et al., 2018). In non-health programs, immature myeloid cells undergo differentiation into granulocytes, macrophages, and dendritic cells (Groth et al., 2019). MDSCs can be divided into two subgroups, including polymorphonuclear MDSCs (PMN-MDSCs) and monocytic MDSCs (M-MDSCs) (Bronte et al., 2016). Additionally, M-MDSCs can restrain T-cell and NK cell activity by secreting reactive oxygen species (ROS), nitrogen oxide (NO), arginase-1, etc., therefore suppressing the immune response of the host (Veglia et al., 2018). In our experiments, increased M-MDSC proliferation to SCI was suppressed by hAMSC treatment, and it might have some positive effects on the recovery of the function. Furthermore, when considering the biology of macrophages, their differentiation and polarity are significant factors to consider; non-lymphocytic Ly6G^−^CD11b^+^F4/80^+^Ly6C^+^ cells were also suppressed significantly, which were categorized as M1 macrophages/microglia (Sica and Mantovani, 2012; Yang et al., 2014) and play a role in initiating acute inflammation (Graubardt et al., 2017; Swirski et al., 2007).

Finally, NO is a diatomic, short-lived gas regulating a wide range of homeostatic functions, primarily in the cardiovascular and nervous systems (Vallance and Charles, 1998). The previous report showed that NO generated in the CNS during inflammation induced neuronal death through glutamate release, leading to excitotoxic cell death. Inhibition of NO has been demonstrated to protect neurons from death, as evidenced in both *in vitro* experiments and animal models (Liy et al., 2021). In our experiments, we observed a significant suppression of NO production in the hAMSC-treated group. This may suggest the potential of hAMSC administration to suppress inflammatory responses and exert neuroprotective effects.

In this study, we have yet to provide evidence of the specific target site of hAMSC action, which warrants further investigation. Additionally, we plan to explore whether hAMSC treatment leads to changes in the number of neurons at the injury site and whether this impacts symptom improvement.

## Conclusions

We have demonstrated that intravenous administration of hAMSCs improved the gait and sensory function in the chronic phase. The improvement may be attributed to the suppression of inflammatory responses. In our experimental model, hAMSC treatment suppressed macrophage accumulation and inflammation locally, suggesting neuroprotective effects. Additionally, systemic effects were indicated by suppressed production of M-MDSCs and M1 macrophages, along with decreased serum NO concentration following hAMSC treatment. These findings suggest potential benefits of hAMSC treatment for SCI patients in the future.

## Data Availability

The datasets used and/or analyzed during the current study are available from the corresponding authors on reasonable request. Requests to access these datasets should be directed to Shoichiro Tsuji, sh-tsuji@hyo-med.ac.jp, and Yoji Kuramoto, yo-kuramoto@hyo-med.ac.jp.
